# Transcriptome Analysis Reveals genes involved in flavonoid biosynthesis and accumulation in *Dendrobium catenatum* From Different Locations

**DOI:** 10.1038/s41598-018-24751-y

**Published:** 2018-04-23

**Authors:** Zhouxi Lei, Chunhua Zhou, Xiaoyu Ji, Gang Wei, Yuechun Huang, Wenxia Yu, Yingyi Luo, Yue Qiu

**Affiliations:** 1 School of Pharmaceutical Sciences, Guangzhou University of Chinese Medicine, Guangzhou city, 510006 China; 2 Research Center of Chinese Herbal Resource Science and Engineering, Guangzhou University of Chinese Medicine, Guangzhou city, 510006 China; 3 The First Affiliated Hospital, Guangzhou University of Chinese Medicine, Guangzhou city, 510006 China

## Abstract

In this study, we applied transcriptome and UHPLC-MS technologies to investigate the flavonoids and their biosynthesis- and accumulation-related genes in *Dendrobium catenatum* from three different locations. Eight flavonoid glycosides were identified using standard references or previously isolated substances with MS data analysis. The total flavonoid contents were determined by reagents, and all the data were analyzed. In total, 23139 unigenes were obtained using the *Dendrobium catenatum* genome data. Of these, 10398 were annotated in the Gene Ontology (GO) database, 4203 were annotated in the KEGG database, and 10917 were annotated in the EuKaryotic Orthologous Groups (KOG) database. Thirty-one of the unigenes annotated by the KEGG database were involved in flavonoid pathways. The genes involved in bio-modification, accumulation, transportation and the regulation of the flavonoid bio-synthesis process were investigated. In conclusion, the flavonoids in *Dendrobium catenatum* from three different locations were different in quantitative and qualitative which may contribute to the establishment of quality control method for this herbal plant. These differences were determined by flavonoids biosynthesis process and they were concluded by sorting out the expression level of certain biosynthesis related genes.

## Introduction

In 2016, the number of the *Orchidaceae* species reached 27753 according to the annual checklist from the Catalogue of Life (http://www.catalogueoflife.org/), which made it the largest family of *Liliopsida*. *Dendrobium*, which is mainly distributed in India, southern Asia, Japan, Australia and some Pacific islands^1^, is the largest genus in the *Orchidaceae* family, with 1527 species worldwide. *Dendrobium* species, especially *Dendrobium catenatum*, are highly prized in folk medicine in China and some South Asian countries^2^. Due to over-excavation and destruction of their natural environment, *Dendrobium catenatum* is almost extinct in the wild^3^. Modern pharmacological studies have revealed that *Dendrobium catenatum* possesses many bioactivities, such as immune modulation, anti-oxidant, anti-inflammation and anti-tumor activities^4–7^. It is commonly accepted that the bioactive compounds in *Dendrobium catenatum* are polysaccharides, alkaloids and flavones^8^. Of these bioactive compounds, polysaccharides are the dominant substances in the *Dendrobium catenatum* stem, which mainly consists of mannose and glucose^9^. Following polysaccharides, flavonoids ranked as the second most common compounds in *Dendrobium catenatum*. Flavonoids were reported to be a large group of secondary metabolites in the plants, which are famous for their anti-oxidant and protective effects on cell toxicity^10^.

There are three major groups of secondary metabolites in plants: phenolics, terpenoids and alkaloids. Flavonoids are the first class of phenolics and are known for their high diversity and wide distribution in plants. Their basic structure is characterized by carbon segments that can be divided into several groups, such as flavanones, isoflavones, flavones, flavonols and anthocyanins^11^. To date, few studies have qualitatively or quantitatively studied flavonoids from the *Dendrobium* genus or their bio-synthesis pathways. Using various retrieval methods, we have identified several reports focused on flavonoid constituents; thus, the basic chemical structure of flavonoids from the genus *Dendrobium* can be concluded^12–14^. First, it has been proven that most flavonoids in *Dendrobium* are C-glycosides, with only few of them being O-glycosides. Second, the skeletons of most flavonoids in *Dendrobium* are vitexin, quercetin, kaempferol, apigenin, etc. Lastly, the flavones can be analyzed by MS by observing characteristic ion fractions and their relative abundance. By studying the flavonoids in plants, it is widely accepted that the precursors of most flavonoids are malonyl-CoA and p-coumaroyl-CoA and that their synthesis begins in the phenylpropanoid pathway^15^. In addition to flavonoid pathway-related enzymes, the bio-synthesis of flavonoid glycosides in plants is also regulated by many transcription factors, such as the MYB-bHLH-WDR complex. And also regulated by UDP-glycosyltransferase and cytochrome P450^15^.

In the last few years, transcriptome technology has become a widely used tool to investigate the bio-synthesis of secondary metabolites. Large numbers of studies have been performed to reveal the bio-synthesis pathways of bio-active constituents in plants. Because flavonoids are a large group of important natural compounds, they have attracted much attention worldwide regarding their bio-synthetic pathways in plants and examining these via transcriptome analysis. It clearly stated that transcriptome analysis is a great tool for secondary metabolic analysis.

The first transcriptome analysis paper focused on *Dendrobium catenatum* was published in 2013. It revealed the alkaloid biosynthetic genes and developed some genetic markers for germplasm breeding and adulterated species identification^16^. In 2016, 4 papers focused on the transcriptome analysis of this orchid herb were published. These studies revealed the regulatory map, gene regulation networks under cold stimulation, the identification of genes associated with polysaccharide synthesis and gene expression analysis of the protocorm^1,17–19^. Also in 2016, the Shenzhen Key Laboratory for Orchid Conservation and Utilization published the *Dendrobium catenatum* genome sequence and revealed its polysaccharide synthesis process^20^. The genome sequence data can be obtained from NCBI. In 2017, a research group at Hangzhou Normal University published a paper on the transcriptome analysis of *Dendrobium catenatum* that compared the expression levels of polysaccharide bio-synthesis pathway-related enzymes in the flower, leaf, stem and root. However, no reports have focused on the flavonoid bio-synthesis pathway in *Dendrobium catenatum*. In this study, we combined constituent and transcriptome analyses to reveal the flavonoid bio-synthesis pathway and related enzymes. And tried to explain the quantitative and qualitative differences of flavonoids in samples from three different locations in bio-informatics way.

## Methods

### Plant material and RNA extraction

**Figure 1:**
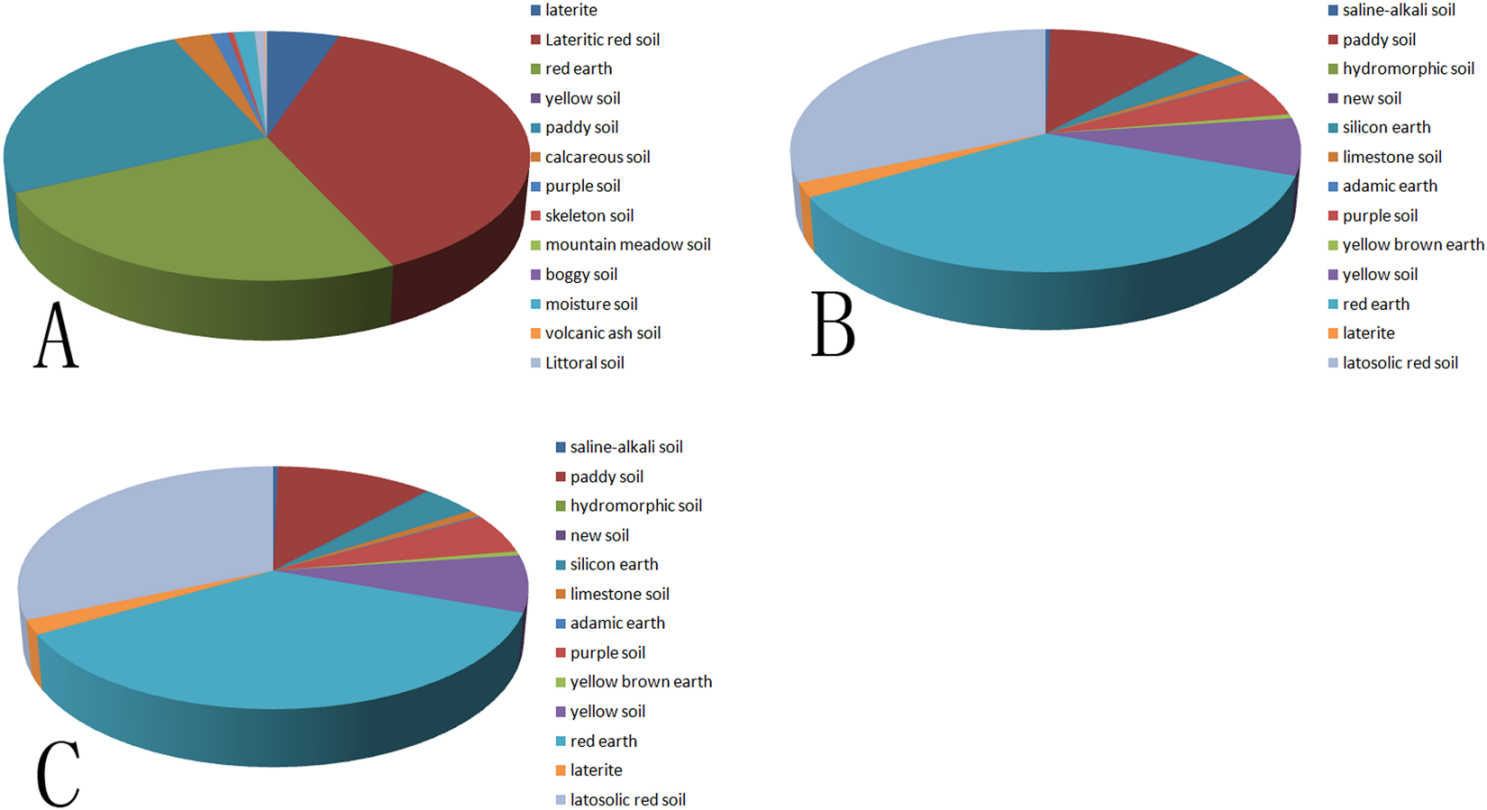
The soil composition of Guangdong (**A**), Guangxi (**B**) and Zhejiang (**C**) provinces.

**Figure 2:**
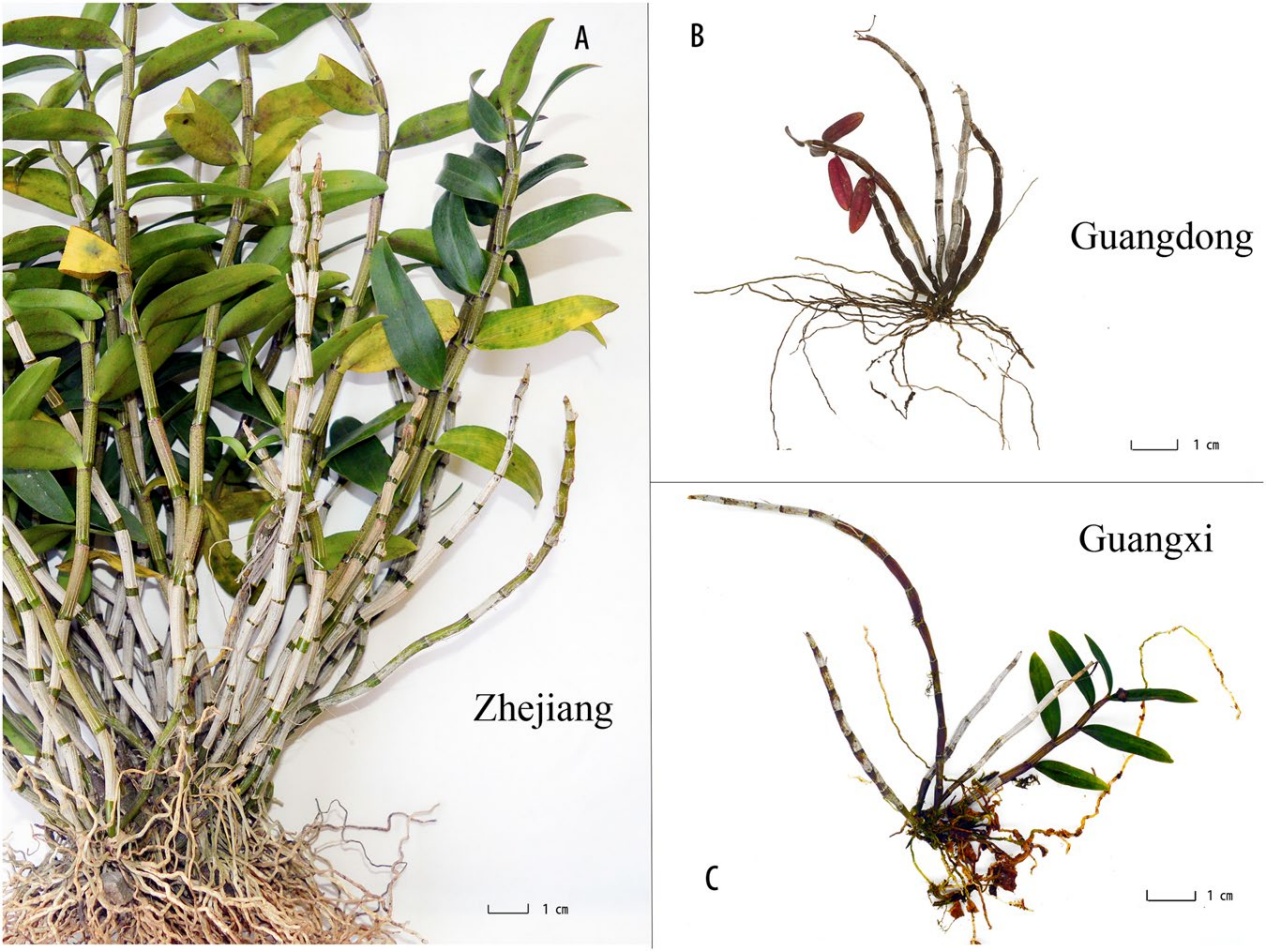
The Dendrobium catenatum samples from Zhejiang (**A**), Guangdong (**B**) and Guangxi (**C**) provinces.

The seeds of *Dendrobium catenatum* from three main representative production locations, namely, Zhejiang province, Guangxi province and Guangdong province in China were collected from their own places of production for breeding. All samples were grown from *Dendrobium catenatum* seeds in a greenhouse at 26 °C, 65–75% humidity and a 12 h/12 h dark/light cycle control. After 6 months, the plants were transferred to the wild to simulate their normal ecology. The climate conditions and soil composition of these three different places were significantly different. The main soil in Guangxi and Guangdong is red earth, which is rich in hematite. The soil composition of Guangdong, Guangxi and Zhejiang was shown in Fig. 1. From the pie chart we can see, the soil of Guangdong was mainly composed of paddy soil, red earth and lateritic red soil. The soil of Guangxi was mainly composed of red earth, latosolic red soil and paddy soil. The soil of Zhejiang was mainly composed of red earth, yellow soil and paddy soil. The Guangxi province belongs to the Tropical Monsoon Climate area, the annual rainfall is the highest in China and the average temperature is 16.0–23.0 °C. The seasonal trend in Guangxi is not distinct. The annual sunshine duration in Guangxi is 1169–2219 hours. The Guangdong province belongs to the Subtropical Monsoon Climate. The annual rainfall is between 1300–2500 mm. The average temperature is 19.0–24.0 °C. The annual sunshine duration in Guangdong is 1500–2300 hours. And Zhejiang province belongs to the Subtropical Monsoon Climate. The latitude of Zhejiang is higher than Guangdong and Guangxi. So the average temperature is 15.0–18.0 °C. It snows in winter and the annual rainfall is 980–2000 mm. The annual sunshine duration in Zhejiang is 1710–2100 hours. Samples for further experiments were collected from 2-year-old plants in spring. As shown in Fig. 2, the external characteristics of these plants differ even though the plants belong to the same species. Three biological replicates retrieved for each sample, and total RNA of the stem was extracted using a TRIzol kit according to the manufacturer’s guideline. The concentration and quality of the extracted RNA was tested by an Agilent 2100 RNA 6000 Nano kit.

### The determination of flavonoid content and UHPLC-MS analysis

The *Dendrobium catenatum* stem was dried in an oven at 60 °C and crushed into powder. In total, 1.0000 g of powder was precisely weighed and transferred to a conical flask. Then, 30 mL of petroleum ether was added to remove the lipids before extracting three times with 50 mL of 80% ethanol ultrasonically. The extract was dried in a water bath and re-dissolved in distilled water. The solution was eluted in D101 Macroporous Resin with distilled water and 95% ethanol. The ethanol elution was collected for flavonoid content determination. The ultraviolet absorption of the ethanol elution was tested in a wavelength of 510 nm. And the total content of flavonoids in the elution was calculated by a standard curve using rutin in different concentration. Three biological repeats were completed in this experiment.

The UHPLC-MS analysis of the flavonoids in *Dendrobium catenatum* was performed as follows: 1.000 g of powder was precisely weighed and transferred to a conical flask; 50 mL of methanol was added, and the mixture was extracted ultrasonically for 45 min. The extraction was filtered by filter paper with an aperture of 80 μm, the filtrate was collected, 50 mL of 80% methanol was added to the residue, and this solution was extracted ultrasonically for 45 min. The extraction was filtered to obtain the filtrate. The filtrate was combined and concentrated with a hypobaric drying method. Eighty percent methanol was used to dissolve the solution to the required concentration to obtain the test solution for UHPLC-MS analysis. The UHPLC-MS analysis was performed by following a previously published method by our research group^13^ with some modification. The flow rate of UHPLC column was 200 μL/min, and the column temperature was 35 °C. The elution gradient was a mixture of methanol and ammonium acetate buffer. MS was performed in negative ion mode. The isolation width was 1.0 m/z, the collision gas was He and the collision energy was 35%. Seven standard references were applied to help identify the chromatography peaks, 5 of which, vicenin I, vicenin II, 6,8-di-C-α-L-arabinosylapigenin, rutin and violanthin, were separated previously, while the other 2, schaftoside and vicenin III, were purchased from the National Institute for the Control of Pharmaceutical and Biological Products.

### cDNA library preparation and transcriptome sequencing

The mRNA was enriched with Oligo(dT) beads (Thermo Fisher Scientific, MA, USA) from the extracted RNA. The purified mRNA was fragmented into short 200–400 bp sequences using fragmentation buffer. The short mRNA fragments were transcribed into cDNA with random primers, purified with a QiaQuick PCR extraction kit and ligated to Illumina sequencing adapters. The ligation products were sequenced using an Illumina Hiseq^TM^ 4000 by Gene Denovo Biotechnology Co. (Guangzhou, China)

### Raw reads filtration, alignment by genome and basic annotation

Before annotation, the raw reads obtained from the sequencing were purified by several procedures including removing reads containing adapters, removing reads containing more than 10% unknown nucleotides and removing low-quality reads containing more than 50% of low-quality bases (Q-value ≤ 10). The basic information for all the sequencing data, including the percentage of duplicate reads, GC content, and modules failed, are shown in the supplementary data. The sequencing data were assessed by FastQC, Per Sequence Quality Scores, Per Sequence GC content and Sequence Duplication Levels. A *Dendrobium catenatum* genome database was used as a reference for alignment and annotation. The expression levels of all the unigenes were presented as the FPKM. The FPKM values for all the samples are presented in the supplemental data after correction.

### Transcript expression, replicate correlation analysis, principal component analysis and differentially expressed gene (DEG) analysis

The expression of the mapped unigenes was calculated and normalized to the FPKM values with the following formula:

$${\rm{FPKM}}=\frac{{\rm{Total}}\,{\rm{exon}}\,{\rm{Fragments}}}{{\rm{mapped}}\,{\rm{reads}}({\rm{million}})\times {\rm{exon}}\,{\rm{length}}({\rm{KB}})}$$

The correlation analysis of the three biological replicates from the three groups was performed to examine the reliability and operational stability of the results. The repeatability between the three replicates increased as the correlation coefficient reaches 1. Principal component analysis was performed with R package models to reveal the relationships between all the data. The identified differentially expressed genes were analyzed based on a fold change ≥ 2 and a false discovery rate (FDR) ≤ 0.05, and p ≤ 0.05 was the threshold to determine significant differences between the three groups. The DEGs were also subjected GO functional and KEGG pathway (http://www.kegg.jp/kegg/kegg1.html) analysis.

## Results

### The total flavonoid contents and UHPLC-MS analysis

**Figure 3:**
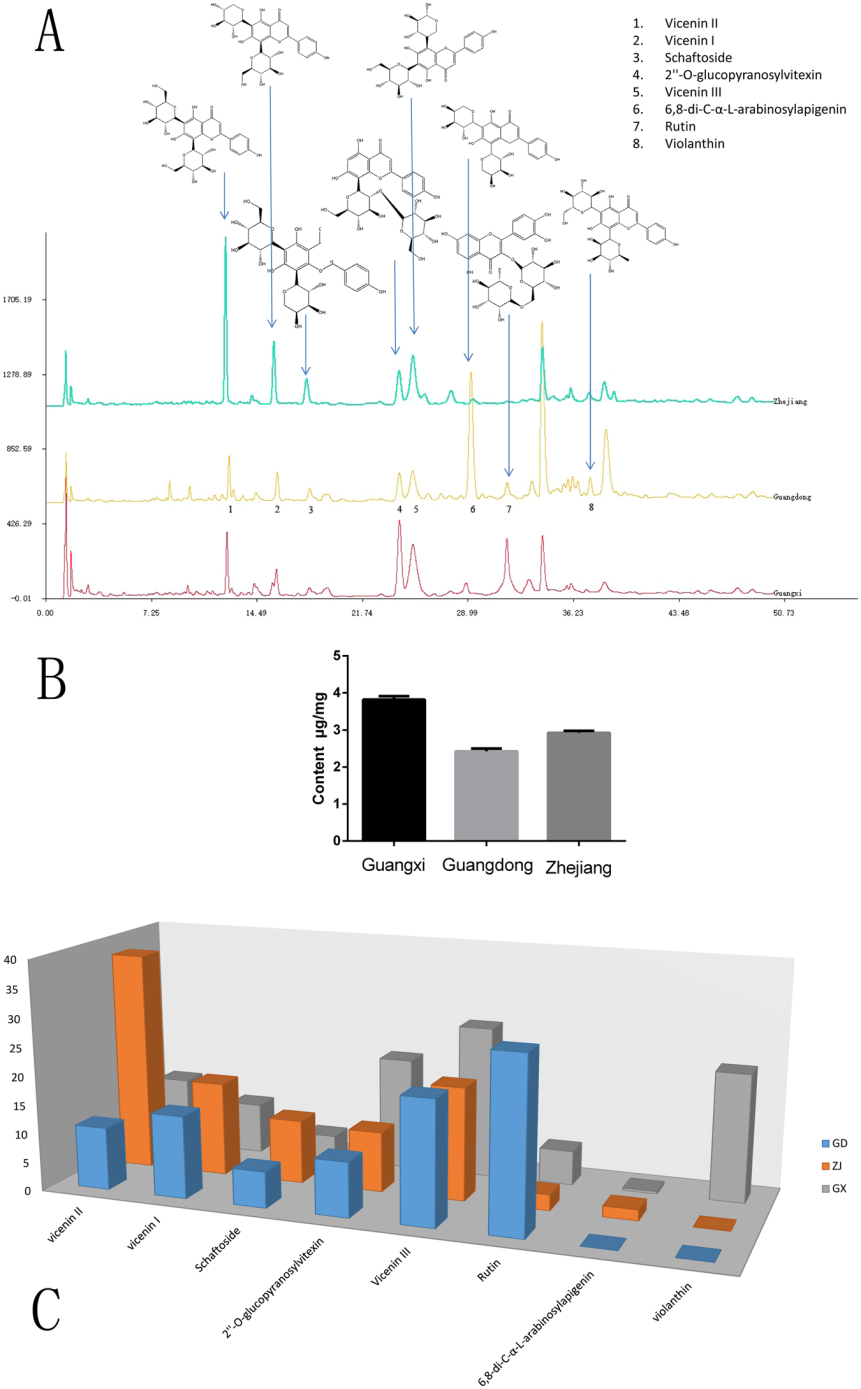
The UHPLC-MS chromatographic (**A**), total flavonoids content (**B**) and the relative content of eight flavonoid glycosides (**C**).

The results showed that the total flavonoid contents in the samples from the three provinces were vastly different from one another. The standard curve and calculation method was shown in supplemental data. The total flavonoid contents in the samples from Guangxi province were the highest, with a content of 3.87 μg/mg, while the total flavonoid contents of the samples from the Guangdong and Zhejiang provinces were lower, 2.40 and 2.85 μg/mg, respectively. Eight flavonoid glycosides were identified from the samples with standard references and by analyzing the ion fragmentation patterns. All the identified flavonoid glycosides except rutin were C-glycosides. The skeletons of all but rutin were apigenin. When comparing the UHPLC-MS data from the samples from the three different production locations, it could be concluded that some common constituents, such as vicenin and schaftoside, were present in all the *Dendrobium catenatum* samples. However, compositional diversity existed between the samples. It was obvious that the peak area of rutin was much higher in the samples from Guangdong than in the samples from the other two places, while the vicenin II contents were very high in the samples from Zhejiang. It was also obvious that samples from Zhejiang contain a small quantity of 6,8-di-C-α-L-arabinosylapigenin, while his compound was not present in the samples from the Guangdong and Guangxi provinces. The results are shown in Fig. 3.

### Sequencing Quality and Mapping Results

**Figure 4:**
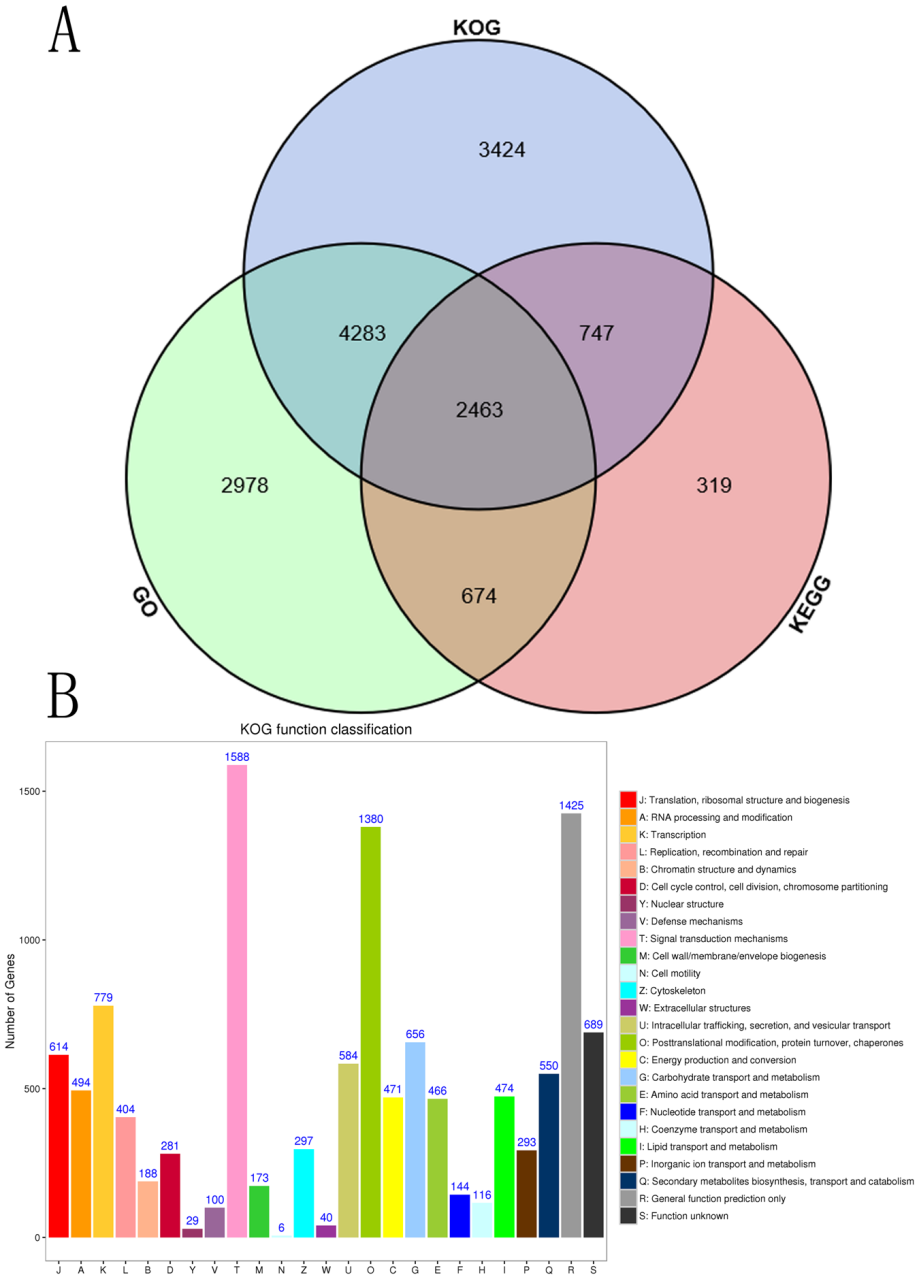
Venn graph of the three database annotation results (**A**) and KOG annotation (**B**).

After sequencing, the number of sequences from all the samples ranged from 13.3 million to 18.2 million. The obtained sequences were processed for quality control analysis. The GC percentage ranged from 45% to 46%. The percentage of duplicate reads ranged from 43.3% to 60.2%. The percentage of modules failed in the FASTQC reports ranged from 8% to 25%. The quality of the sequencing was further checked based on the GC Content Distribution, Mean Quality Scores, Per base N content and Adapter content. All FastQC graphics are shown as supplemental data. After quality control, the sequences were processed for mapping. In total, 23139 unigenes were obtained. The expression levels, correlation analysis and principle component analysis of the 23139 unigenes are shown in the supplemental materials. Of the 23139 unigenes, 10398 were annotated by the GO database, while 4203 were annotated by the KEGG database. Blasting the unigenes lead to 10917 of them being annotated by the KOG database. The venn graph of the three database annotation results is shown in Fig. 4A. From the graph, we can see that 2463 unigenes were annotated by the GO, KEGG and KOG databases simultaneously. When combining the number in the venn graph, there were 14888 unigenes that were annotated by at least one of the three databases, which is approximately 64.3% of all the mapped unigenes.

### KOG annotation of the mapped unigenes

Based on the KOG annotation, most unigenes (1588) were annotated as Signal transduction mechanism. Additionally, 1425 unigenes were assigned to the General function prediction only classification. In total, 1380 unigenes were annotated as Posttranslational modification, protein turnover, and chaperones., Moreover, 550 unigenes were annotated as Secondary metabolites bio-synthesis, transport and catabolism classification. The results of KOG annotation was shown in Fig. 4B.

### Gene Ontology Classification

**Figure 5:**
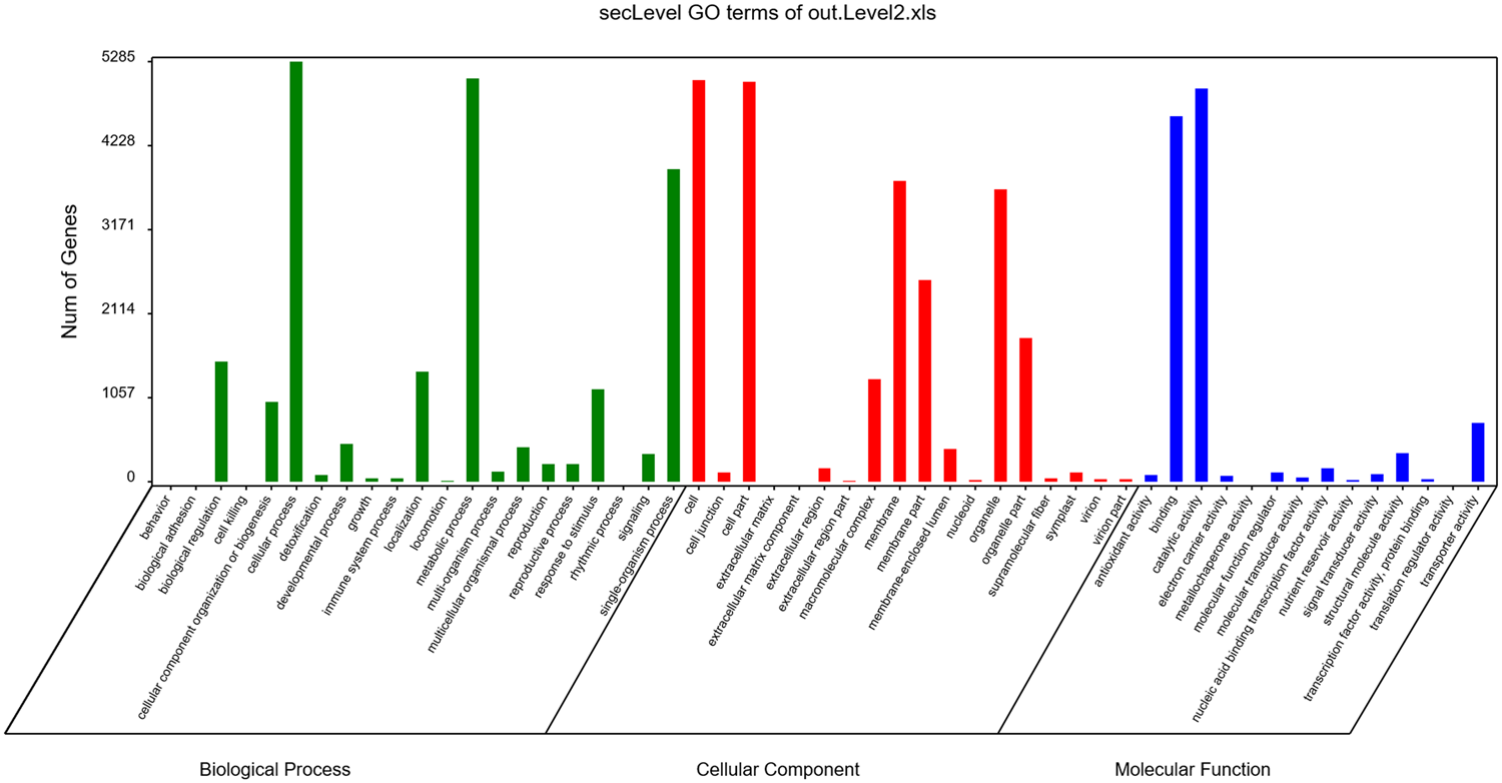
The GO terms of all annotated unigenes.

In the GO classification analysis, 10398 unigenes were annotated and grouped into the 3 basic GO terms: Biological Process, Cellular Component and Molecular Function. In Biological Process, 5285 unigenes were grouped as cellular process, while 5077 unigenes were assigned to metabolic process. Organic substance metabolic process ranked third, with 4079 unigenes. In Cellular component, most of the unigenes were classified as membrane, with 3783 unigenes. The Membrane-bound organelle term ranked second, with 3343 unigenes, and Intracellular membrane-bound organelle ranked third, with 3329 unigenes. When examining the Molecular Function terms, it was clear that most of the unigenes were grouped as binding and catalytic activity. The result of GO classification analysis was shown in Fig. 5.

### Identification and annotation of differentially expressed genes (DEGs)

**Figure 6:**
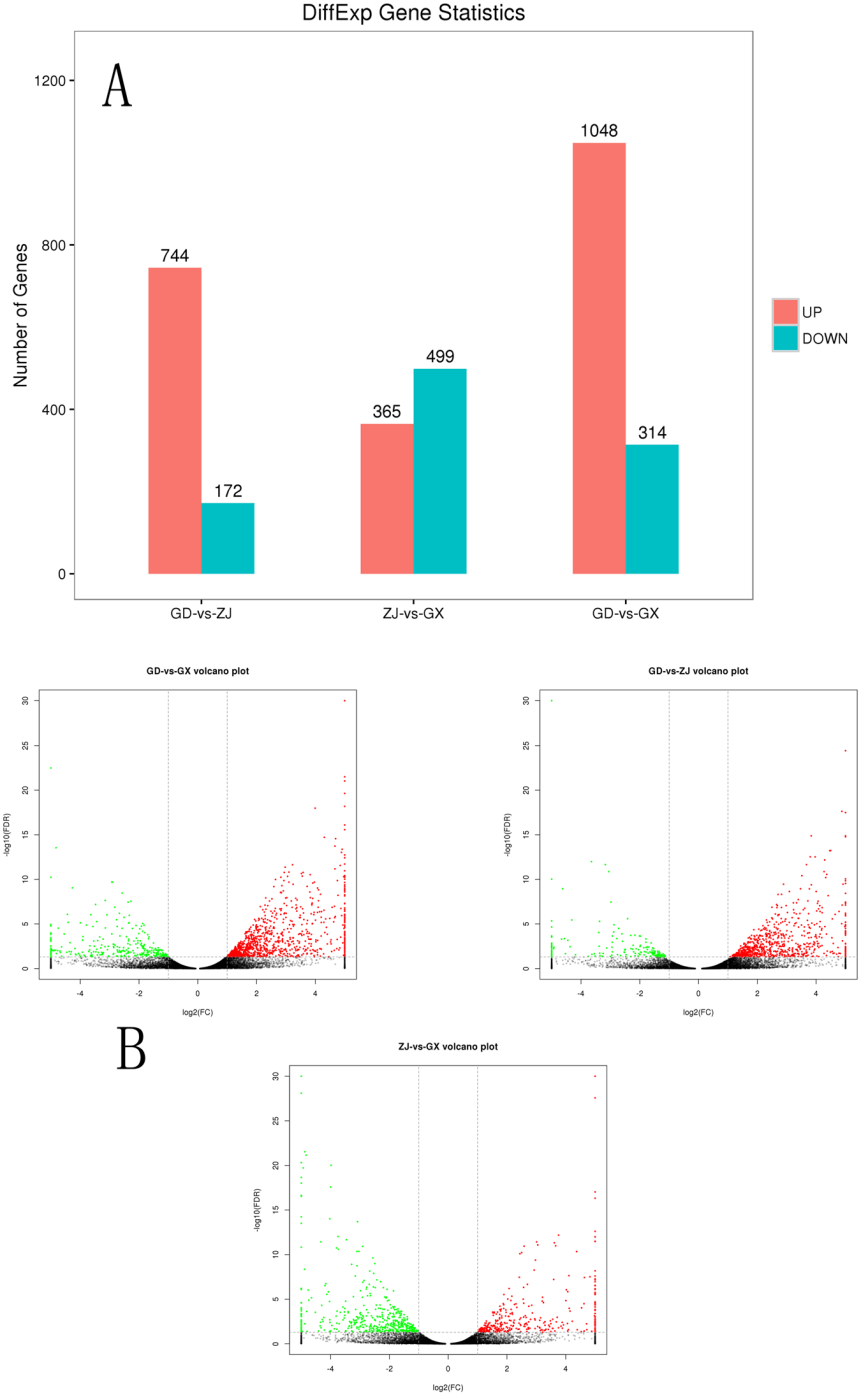
The different expression genes counts (**A**) and volcano plot (**B**).

To identify the differentially expression genes (DEGs) from the samples from the three provinces, the FDR was used as a threshold; it was set as 0.05 for the DEG analysis. When comparing the expression levels of the unigenes from the samples from the Guangdong and Zhejiang provinces, 744 genes were up-regulated, while 172 genes were down-regulated when comparing samples from Guangdong and Zhejiang provinces. In total, 39 of the 744 up-regulated genes and 10 of the 172 down-regulated genes were assigned as secondary metabolites related in the KOG database. Seven CYP450 genes, 4 MYB genes and 11 bHLH genes were found among these DEGs. Comparison of the samples from the Guangdong and Guangxi provinces identified 1362 DEGs, including 314 down-regulated DEGs and 1048 up-regulated DEGs. Forty-one of the up-regulated genes were annotated by the KOG database as secondary metabolites bio-synthesis, while 21 of the down-regulated genes were annotated as the same classification. Among these 1362 DEGs, 14 were CYP450 genes, including CYP71, CYP78, CYP86 and CYP94. Ten MYB genes, 8 bHLH genes and 1 WD40 gene were also identified. These DEGs may play an important role in flavonoid bio-synthesis in plants. Comparison of the samples from the Zhejiang and Guangxi provinces detected 864 DEGs; 365 were up-regulated, and 499 were down-regulated. Seventeen of the up-regulated genes were assigned as secondary metabolites related, and Thirty seven of the down-regulated genes were assigned as the same term by the KOG database. Twelve CYP450 genes, including CYP71, CYP87, CYP86, CYP90 and CYP98, were detected. Moreover, 3 MYB transcription factors and 5 bHLH were also identified. The result of DEGs was shown in Fig. 6.

### Candidate genes involved in flavonoid bio-synthesis

**Figure 7:**
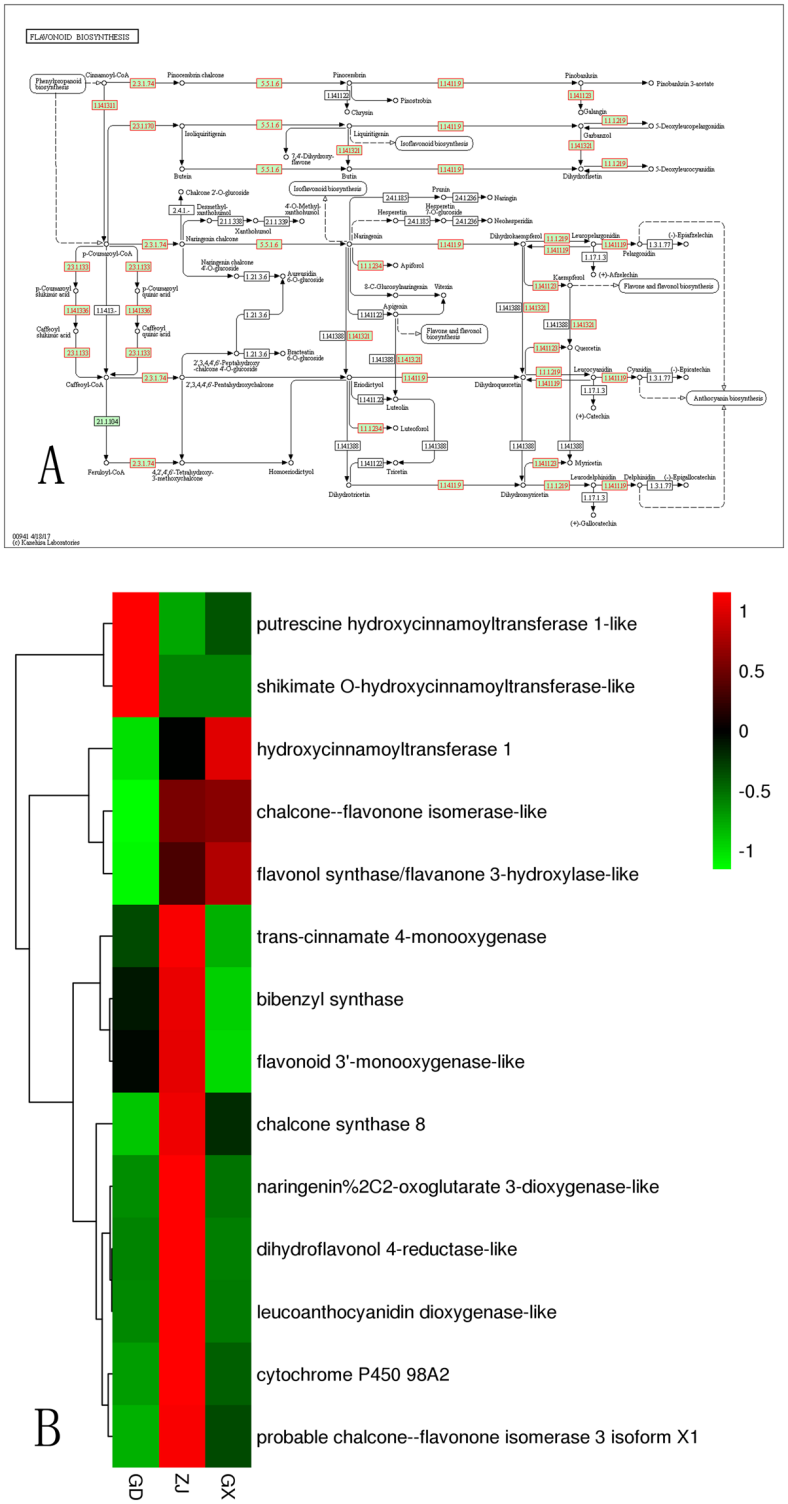
The flavonoids KEGG bio-synthesis pathway^21^ (**A**) in *Dendrobium catenatum* (http://www.kegg.jp/kegg/kegg1.html) and the heatmap of related genes (**B**).

The HPLC-MS analysis confirmed that the main alcohol-soluble components in *Dendrobium catenatum* were flavonoid glycosides with the following three basic skeletons: quercetin, luteolin and apigenin. To date, no research has been performed to study the flavonoid biosynthesis pathway in *Dendrobium catenatum*. After mapping, 4203 unigenes were annotated by the KEGG database (http://www.kegg.jp/kegg/kegg1.html). Among the 4203 unigenes, 31 belonged to the flavonoid biosynthesis pathway. These 31 genes encoded 14 enzymes in this pathway. The expression levels of the genes encoding these 14 enzymes are shown in Fig. 6. From Fig. 6, we can see that these 31 annotated unigenes included all the genes from the *Dendrobium catenatum* genome in the flavonoid biosynthesis pathway. The expression levels of most of these genes were higher in the samples from Zhejiang than in the samples from the other two provinces. The flavonoid biosynthesis pathway showed that the three basic flavonoid glycoside skeletons are regulated by FLS, CYP75A, and flavonoid 3′-monooxygenase. The function of FLS is to regulate the synthesis of kaempferol from dihydrokaempferol; it also transforms dihydroquercetin to quercetin. Kaempferol can also be transformed into quercetin through catalysis by CYP75A and flavonoid 3′-monooxygenase. CYP75A and flavonoid 3′-monooxygenase can also regulate the synthesis of luteolin from apigenin. However, CYP75A from *Dendrobium catenatum* was not annotated by the KEGG database. Additionally, CYP75A was not identified in the annotation from the other databases. Thus, the biosynthesis of these three basic skeletons must be regulated by other cytochrome P450 enzymes or only regulated by FLS (flavonol synthase) and flavonoid 3′-monooxygenase. FLS expression levels were high in the samples from Guangxi and Zhejiang. However, 3′-monooxygenase expression levels were higher in the samples from Zhejiang. The expression levels of these two genes were low in the samples from Guangdong, which may be related to the flavonoid contents in these samples. Searching the samples for the flavone and flavonol biosynthesis pathway showed no annotations. The synthesis of flavonoid glycosides from flavonoids was determined to be regulated by glycosyltransferases. Because there was no annotation in the KEGG database, glycosyltransferases would be obtained from other databases. In addition to FLS and flavonoid 3′-monooxygenase, the enzymes that synthesize precursors, such as naringenin, eriodictyol, and naringenin chalcone, could also play an important role in the bio-synthesis of these three basic skeletons. The results of pathway annotation and expression were shown in Fig. 7.

### Candidate genes involved in flavonoid bio-modification and accumulation

**Figure 8:**
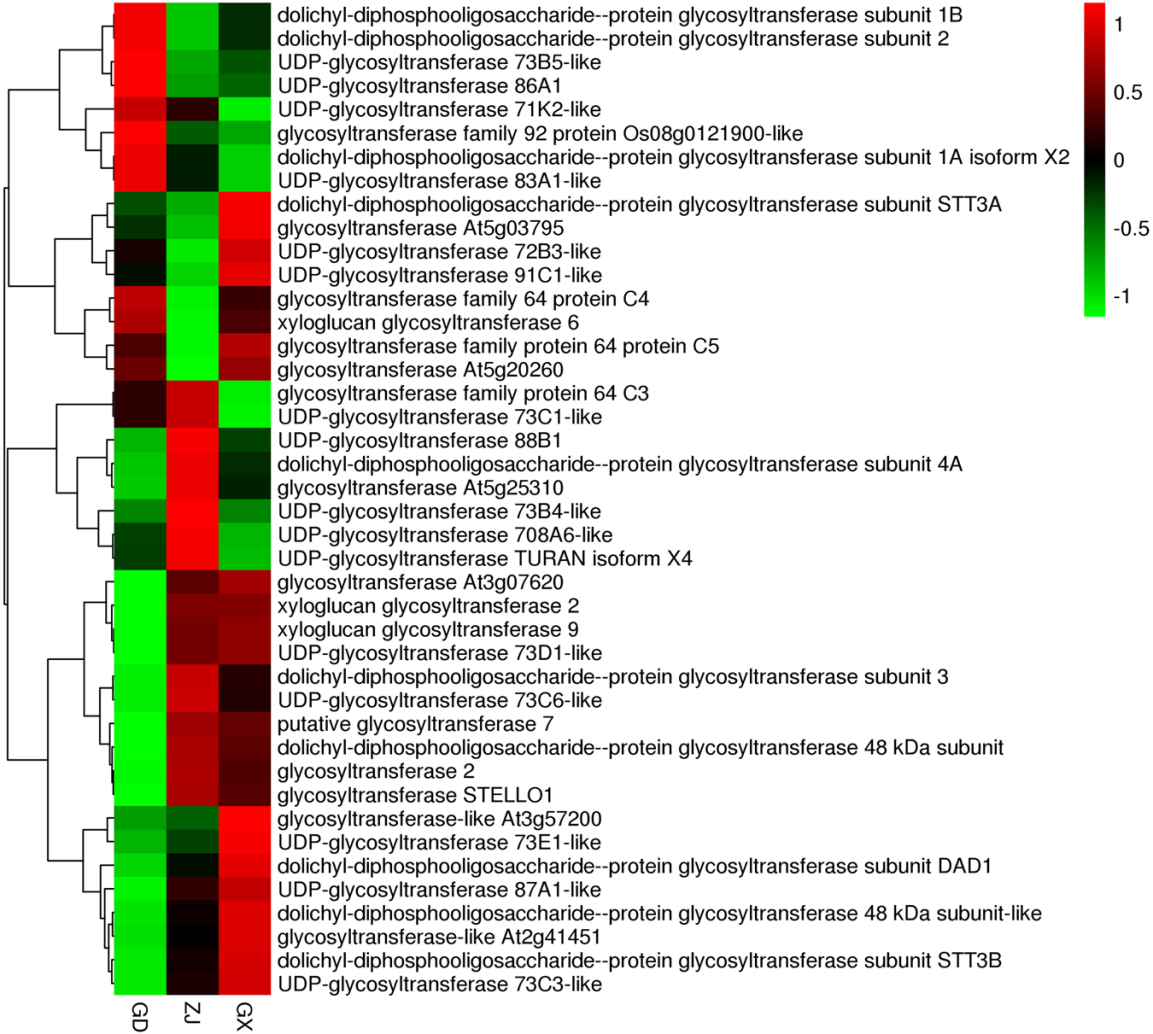
The expression level of all glycosyltransferase genes.

The types and contents of flavonoids in plants depend not only on the flavonoid bio-synthesis pathway, which synthesizes basic aglycones associated with flavonoid glycosides, but also depends on bio-modification and transportation processes. Glycosyltransferases may play an important role in flavonoid bio-modification. In this study, 72 glycosyltransferases were annotated. Among the 72 glycosyltransferases, 42 were annotated by the GO database, 10 were annotated by the KEGG database, and 57 were annotated by the KOG database. The expression levels of the glycosyltransferases are shown in Fig. 8. From the DEGs we can find that there were 12 unigenes encoding glycosyltransferase exhibit significant different expression pattern. 7 of them belongs to the UGT (UDP-dependent glycosyltransferase) family, they were UGT 73, UGT 89 and UGT 83. From the former study of glycosyltransferases it can be told that Glycosylation of flavonoids is mediated by UGT^21^. There is a wild range of UGTs in plants, but the specific function of the DEGs still required further investigation.

**Figure 9:**
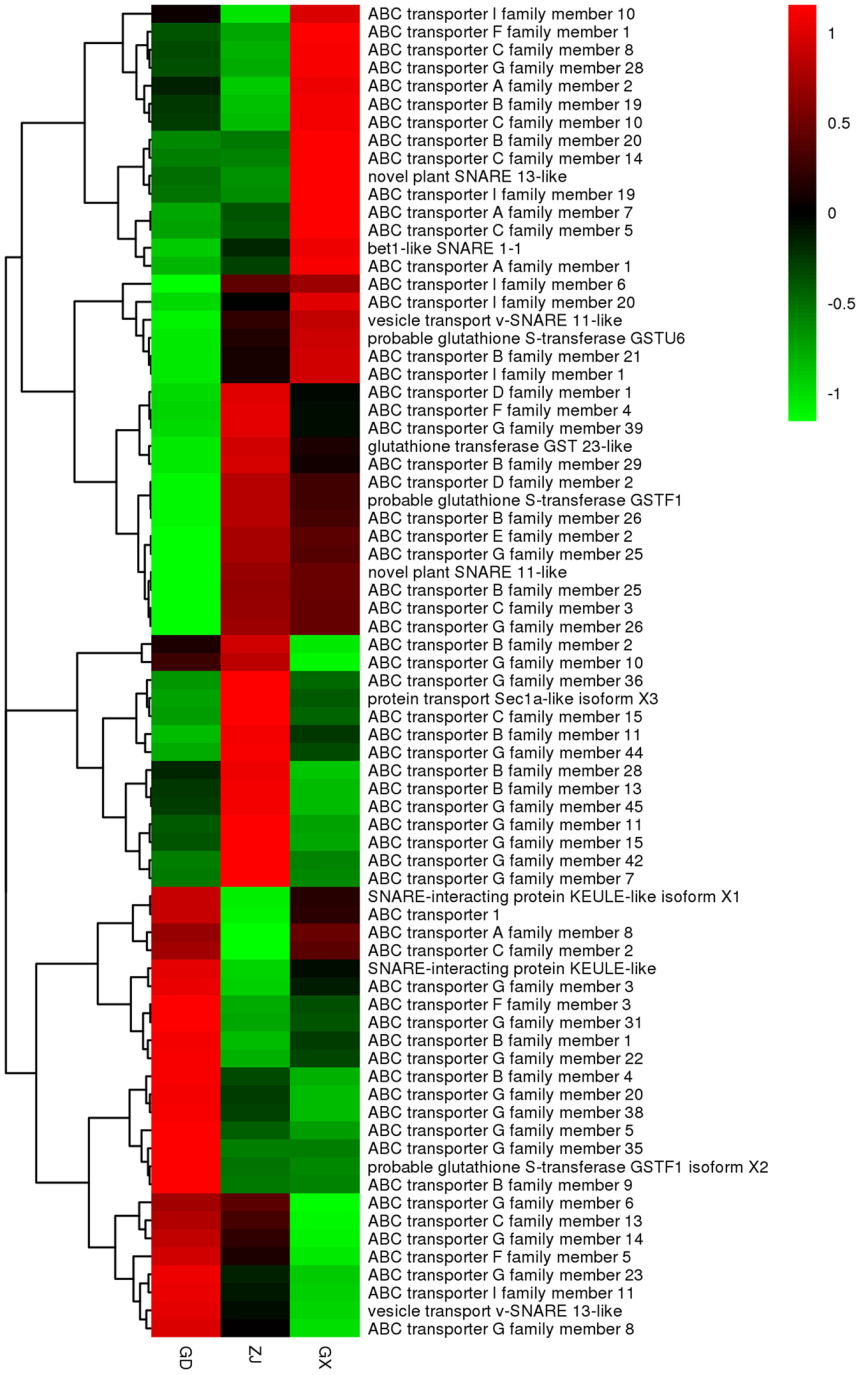
The expression level of ABC transporter, SNARE and GST genes.

It has been reported that some distinctive transporters, including the ABC transporter, SNARE, and GST, play important roles in flavonoid transportation in plants. Analysis of the annotation data detected 115 ABC transporter unigenes, 13 SNARE unigenes and 8 GST unigenes. From the DEGs we can find that the expression level of 12 ABC transporters was significantly different from each other. The ABC transporter was characterized as transporting plant hormones^22^, and also claimed to sequestration flavonoids into vacuole^23^. The expression level of ABC transport represented this ability. However the relation between the expression of the exact ABC transporter genes and flavonoids content still require further study. Only 1 of the 13 SNARE unigenes characterized as SNARE-interacting protein KEULE was found in the DEGs. In plants, SNARE involved in vesicle-mediated secretion of exocytosis and endocytosis^24^. However, the direct role of the SNARE in delivering flavonoids to vacuole was still not demonstrated. Further investigation has to be done^25^. All 8 GST unigenes were annotated by at least one of the three basic databases (GO, KEGG or KOG). After searching the DEGs, 1 of the 8 GST unigenes characterized as glutathione transferase GST 23 showed different between samples. The role of GST in plants was to be the carrier of flavonoids to transport^25^. A report showed that the expression of GST would be related to the accumulation of anthocyanin^26^. The expression levels of glycosyltransferase, ABC transporter, SNARE and GST are shown in Fig. 9.

### The P450 Family Gene Expression

**Figure 10:**
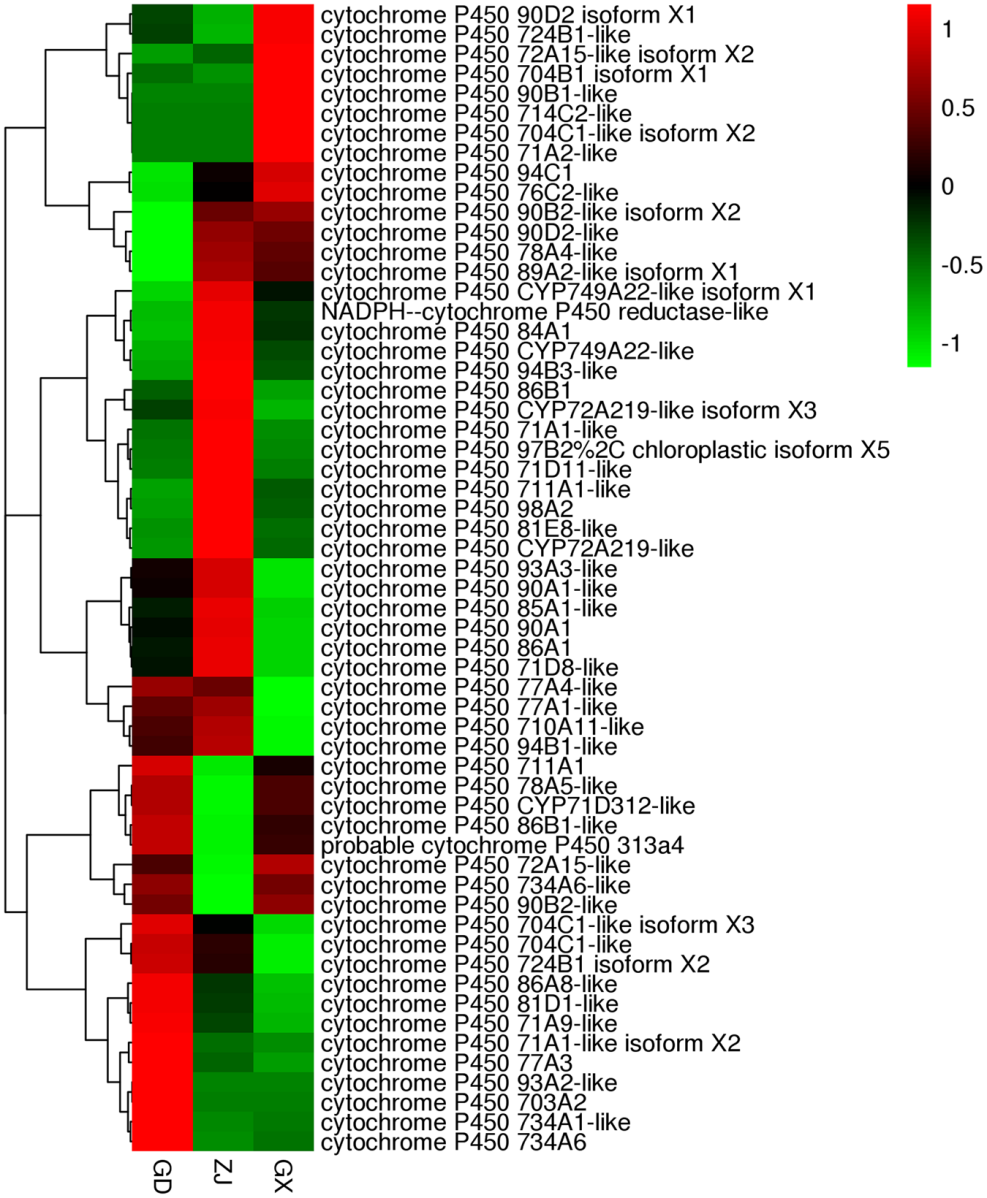
The expression level of CYP450 genes.

P450 enzymes involved in the generation of defense secondary metabolites like flavonoids^27^. Since flavonoids were characterized as defensive compounds, the expression pattern of P450 has a great impact on the flavonoids content in plants^28^. In this study, 133 unigenes were annotated as putative cytochrome P450 members, and 111 of them could be grouped into 53 CYP subfamilies. Twenty-seven of these 133 unigenes were grouped as CYP 71 A1, while 7 were grouped as CYP 89A2. The number of unigenes grouped as CYP 704C1, CYP 71D7 and CYP 78A5 were 5. Among the 133 unigenes, the expression level of 14 unigenes was 0. Through searching the DEGs, the expression level of 20 from the 133 unigenes was significantly different. 11 of them were characterized as cytochrome P450 71 family. And these 11 P450 genes can divided into 2 subfamilies. 7 of them belong to the CYP 71A and 3 of them belong to the CYP 71D. 2 of them belong to the CYP 86 A and 2 of them belong to the CYP 86B. The others were annotated as CYP 87 A, CYP 90 A, CYP 94 C and CYP 98 A. The expression level of CYP 71 family was related to the heat and cold stress in plants. Some would up-regulated and others would down-regulated in different plant species^29–31^. CYP 86 A in Arabidopsis was reported to involved in suberin monomer biosynthesis^32^. The CYP 98 family was reported to catalyze the 3-hydroxylation in phenylpropanoid pathway. So the expression level of CYP 98 would have a greater effect in the bio-synthesis of flavonoids in plants^33^. The expression pattern of the CYP genes was shown in this study. However, the deeper investigation of the exact role of each CYP genes and the specific flavonoids still require further study. A heatmap was built using the 119 CYP unigenes whose expression levels were greater than 0. This heatmap is shown in Fig. 10.

### Expression of flavonoid bio-synthesis and transport-related transcription factors

**Figure 11:**
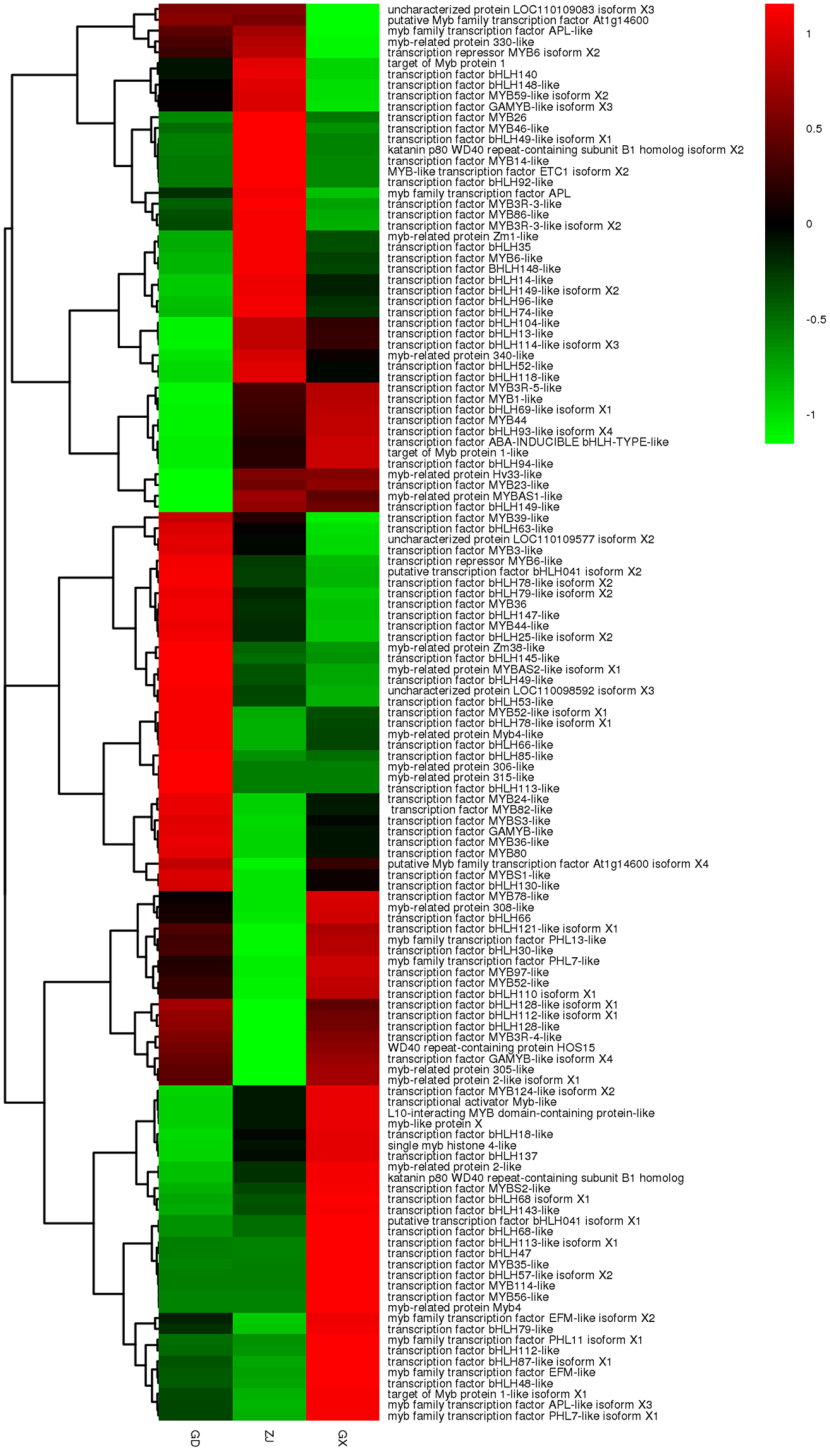
The expression level of genes encoding MYB-bHLH-WD40 complex.

Transcription factors are a critical group of proteins that participate in many biosynthesis processes in living organs. Transcriptome sequencing can provide a detailed view of the regulation of biosynthesis in plants by analyzing TF expression levels. In total, 681 genes encoding transcription factors were annotated. Among these TFs, there is a protein complex that consists of MYB and bHLH TFs with a repeat protein WD40, which plays a critical role in initiating different secondary biosynthesis pathway in plants. As it was reported in previous studies, MYB TFs was involved in regulating the metabolism of phenylpropanoid, including flavonoids and anthocyanins^34–37^. In this study, 153 unigenes were found to encode MYB, while 80 unigenes encoded bHLH. Apart from the TF families, 3 unigenes were found to encode WD40 repeat protein. The MYB-bHLH-WD40 complex was the main transcriptional regulators in plants that regulate the biosynthetic pathway of flavonoids^38^. The expression levels or patterns of these three proteins may be related to flavonoid bio-synthesis. In model plant Arabidopsis, some specific MYB genes were identified to be the regulators of flavonoids^39^. In this study, 13 genes from DEGs were found to be annotated as MYB related genes, encoding MYB78, MYB4, MYB44, MYB46 and other MYB dominant protein. Amount these 4 DEGs, the MYB4 was reported to negatively regulate the transcription of cinnamate 4-hydroxylase (C4H) in *Arabidopsis*^40^. And C4H was an important enzyme, the p-cinnamoyl-CoA was synthesized from cinnamoyl-CoA by C4H. 15 DEGs encoding bHLH was found in this study. These DEGs was annotated as bHLH 13, 14, 18, 30, 35, 48, 49, 63, 78, 85, 92, 93 and 96. It was reported that bHLH type (TT8, GL3 and EGL 3) interact to activate anthyocyanin synthesis in plants. However, no studies had been published to study the exact function of these bHLH related genes. The function of the DEGs in this study still required further investigation. The expression level of this complex was shown in Fig. 11.

## Discussion

In recent years, the transcriptome has become a widely used way to study bio-synthesis mechanisms in plants. In 2017, 576 papers were published using transcriptome analysis, though only two studies were mainly focused on flavonoid metabolism in plants. A research group from Sichuan Agricultural University has applied transcriptome analysis for a flavonoid investigation in buckwheat^41^. Another research group from Malaysia reported candidate genes involved in phenylpropanoid and flavonoid metabolism in *Polygonum minus*^42^. The flavonoid bio-synthesis pathway can be drawn, and the expression levels of the related enzymes can be calculated. By combining constituent and transcriptome analyses, the mechanism of flavonoid bio-synthesis can be revealed^43,44^. Flavonoids are synthesized by the phenylpropanoid and polyketide pathways, which consist of enzymes such as PAL, C4H, and 4CL^45^. The starting point for all flavonoids is malonyl-CoA and p-coumaroyl-CoA. Most flavonoids are synthesized by CHS and CHI condensation; F3H, F3′H or F3′5′H oxidation effects; or DFR and LDOX catalysis^25^. In addition to the flavonoid synthesis pathway, the contents and types of flavonoids in plants are regulated by many other proteins, such as transcription factors such as the MYB-bHLH-WD40 complex^46^. Flavonoids in plants exhibit diverse biological functions, including protection against ultraviolet radiation, phytopathogen infection signaling nodulation, enhancing nutrient retrieval, etc^47^, and involve a variety of proteins, including cytochrome P450^48^, UDP-glycosyltransferases (UGTs)^49^, ATP binding cassette (ABC) transporters^50–52^, soluble N-ethylmaleimide-sensitive factor attachment protein receptors (SNARE)^25^, and glutathione S-transferase (GST)^25,53^.

In this study, we selected *Dendrobium catenatum* samples from three different locations to explore the quantitative and qualitative differences among them. We tried to explain these differences based on transcriptome analysis. The total flavonoid contents from the samples from the 3 locations were different. The contents in the sample from Guangxi were the highest, followed by the Zhejiang sample, and then the Guangdong samples. Next, a UHPLC-MS chromatography system was used to explore the quantitative and qualitative differences between the samples. The relative contents of the 8 characterized flavonoids glycosides from the samples showed that 3 specific compounds were prominent. The relative vicenin II content was 37.96% in the samples from Zhejiang, which was much higher than that in the other two provinces (10.89% and 12.19%). Second, the relative rutin contents in the samples from Guangdong were significantly higher than the other two provinces. Finally, violanthin was only present in the samples from Guangxi. It can be speculated that *Dendrobium catenatum* from the Guangdong, Guangxi and Zhejiang provinces can be distinguished by the detection of these 3 specific flavonoid glycosides. The relative contents of the other 5 flavonoid glycosides were approximately the same between the samples.

To study the relationship between the flavonoid bio-synthesis genes and the flavonoid glycoside contents in *Dendrobium catenatum*, we investigated the expression levels of the related genes. In this study, we have revealed the flavonoid bio-synthesis pathway in plant *Dendrobium catenatum*. The synthesis of flavonoids started from cinnamoyl-CoA and p-cinnamoyl-CoA, ended with apigenin, kaempferol and quercetin. Most of the genes in the flavonoid synthesis pathway were highly expressed in the samples from Zhejiang. However, the total flavonoid contents were not the highest in the samples from Zhejiang. The basic skeleton of all the characterized flavonoids glycosides except rutin was apigenin. Thus, it can be speculated that the expression levels of genes involved in apigenin bio-synthesis would determine the total flavonoid contents. Based on the flavonoid synthesis pathway, apigenin was synthesized from narigenin, and narigenin was synthesized from narigenin chalcone via chalcone-flavonone isomerase-like. The heatmap confirms that the expression levels of chalcone-flavonone isomerase-like were high in the samples from both Zhejiang and Guangxi. Therefore, it is clear that the total flavonoid contents correlated with other enzymes. Based on the pathway, other enzymes such as flavonoid 3′-monooxygenase-like also played a role apigenin synthesis. Flavonoid 3′-monooxygenase-like can transform apigenin to luteolin by enzymatic action. The expression levels of flavonoid 3′-monooxygenase-like in the samples from Zhejiang were obviously higher than the samples from Guangxi. We speculate that the expression of flavonoid 3′-monooxygenase-like could explain why the total flavonoid contents in the samples from Zhejiang were lower than the samples from Guangxi, even though most of the genes involved in the flavonoid bio-synthesis pathway were highly expressed in the samples from Zhejiang. In addition to the total flavonoid contents, UHPLC-MS chromatography also showed that the flavonoid glycoside concentrations in the samples from the three different locations exhibited certain differences. For example, the relative vicenin III content was exceptionally high in the sample from Zhejiang, the relative rutin content was high in samples from Guangdong, and the relative violanthin content was only present in the samples from Guangxi. The bio-synthesis process in plants is quite complicated. The synthesis of one particular compound may be related to multiple genes. Thus, it would be difficult to obtain the absolute correlation between genes and specific compounds. We can only assume based on the function and expression levels of the genes on a particular component. However, identifying flavonoid bio-synthesis-related genes from the DEGs will allow for the relationships between specific genes and certain components to be revealed.

Apart from flavonoid bio-synthesis pathway, some specific gene family also played an import role in synthesis as well as regulation in flavonoid synthesis in plants. In this study we focused on glycosyltransferases, ABC transporter, SNARE, GST, CYP450 and MYB-bHLH-WD40 complex. Glycosyltransferases are involved in the glycosylation process of flavonoid glycosides and determine the number or type of glucoses in the glycosides. The ABC transporter, ATP binding cassette (ABC) transporters play an important role in sequestering flavonoids into vacuoles^50–52^. SNARE, short for soluble N-ethylmaleimidesensitive factor attachment protein receptors, has been reported to function in budding, targeting, docking, fusion and recycling in the flavonoid accumulation processes^25^. And glutathione S-transferase (GST) is an important protein for flavonoid transportation^25,53^. Cytochrome P450 proteins also play an important role in regulating the bio-synthesis of secondary metabolites in plants^48^. Finally, the MYB-bHLH-WD40 complex greatly contributes to regulating the bio-synthesis of flavonoids in plants^54,55^. From the results we can see, the expression level of these gene family exhibit different expression pattern amount samples. The results of this study are based on the expression level differences and published data on flavonoid accumulation- and regulation-related genes. These differences may be caused by the environment or epigenetic background. It was clearly demonstrated that the *Dendrobium catenatum* from these three different places of production differ from each other in transcriptome and composition level. The total flavonoids content was different and some compounds exist only in samples from certain places of production as it was stated in the results. Since *Dendrobium catenatum* is a high value medicine in China. These differences would eventually result in pharmaceutical differences. So this investigation was to inform researchers that *Dendrobium catenatum* is a plant with complicate genetic background. The places where the plant samples from would have a great impact in composition analysis or pharmaceutical experiments. However, the relation between pharmaceutical differences and the real roles of these genes involving in bio-synthesis, accumulation, transportation and regulation of flavonoids in plants in flavonoid bio-synthesis and accumulation stil require further investigation.

## Electronic supplementary material

Supplementary Dataset
