# Supplementary material for: Transcriptome Analysis Reveals genes involved in flavonoid biosynthesis and accumulation in Dendrobium catenatum From Different Locations
Source: Sci Rep. 2018 Apr 23;8:6373. doi: 10.1038/s41598-018-24751-y (PMC5913234; doi:10.1038/s41598-018-24751-y)
Supplement: Supplementary file 1 — Supplementary Dataset [file 41598_2018_24751_MOESM1_ESM.doc]

**Transcriptome Analysis Reveals genes involved in flavonoid biosynthesis and accumulation in *Dendrobium catenatum* From Different Locations**

**Zhouxi Lei^1, §^, Chunhua Zhou^1, §^, Xiaoyu Ji^2^, Gang Wei^1, *^, Yuechun Huang^3^, Wenxia Yu^1^, Yingyi Luo^1^, Yue Qiu^1^**

In order to establish standard curve for calculating the total content of flavonoids in *Dendrobium catenatum* from three different locations, the rutin was applied as standard references. .

The standard reference rutin was weight precisely and dissolved in 95% methanol to obtain standard reference in different concentration (0.01, 0.02, 0.05, 0.1, 0.2, 0.3 mg/mL). The absorption of these references was tested in the wavelength of 510 nm with three technical repeats. And the standard curve was established by the mean of the absorption of every concentration. The result was shown in table S1. The formula and R^2^ was shown in Figure S1.

Table S1 The concentration and absorption of standard reference.

| concentration(mg/mL) | absorption1 | absorption2 | absorption3 | mean |
| --- | --- | --- | --- | --- |
| 0 | 0 | 0 | 0 | 0 |
| 0.01 | 0.063 | 0.062 | 0.063 | 0.063 |
| 0.02 | 0.126 | 0.127 | 0.125 | 0.126 |
| 0.05 | 0.337 | 0.336 | 0.337 | 0.337 |
| 0.1 | 0.668 | 0.667 | 0.668 | 0.668 |
| 0.2 | 1.323 | 1.323 | 1.323 | 1.323 |
| 0.3 | 2.006 | 2.007 | 2.006 | 2.006 |


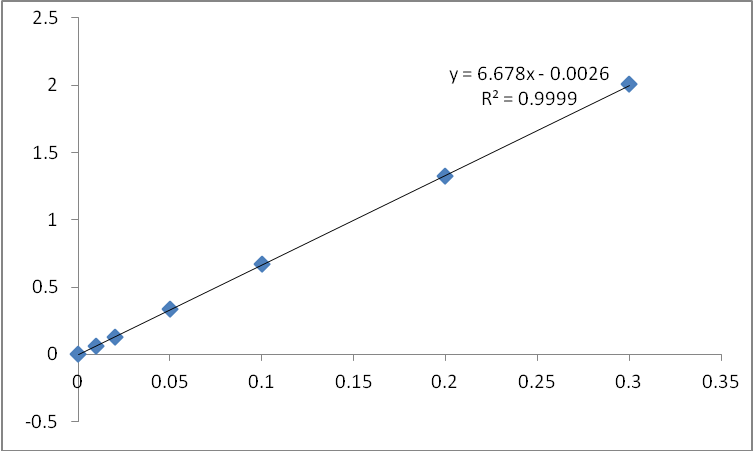


Figure S1 The standard curve, formula and R^2^ of standard reference


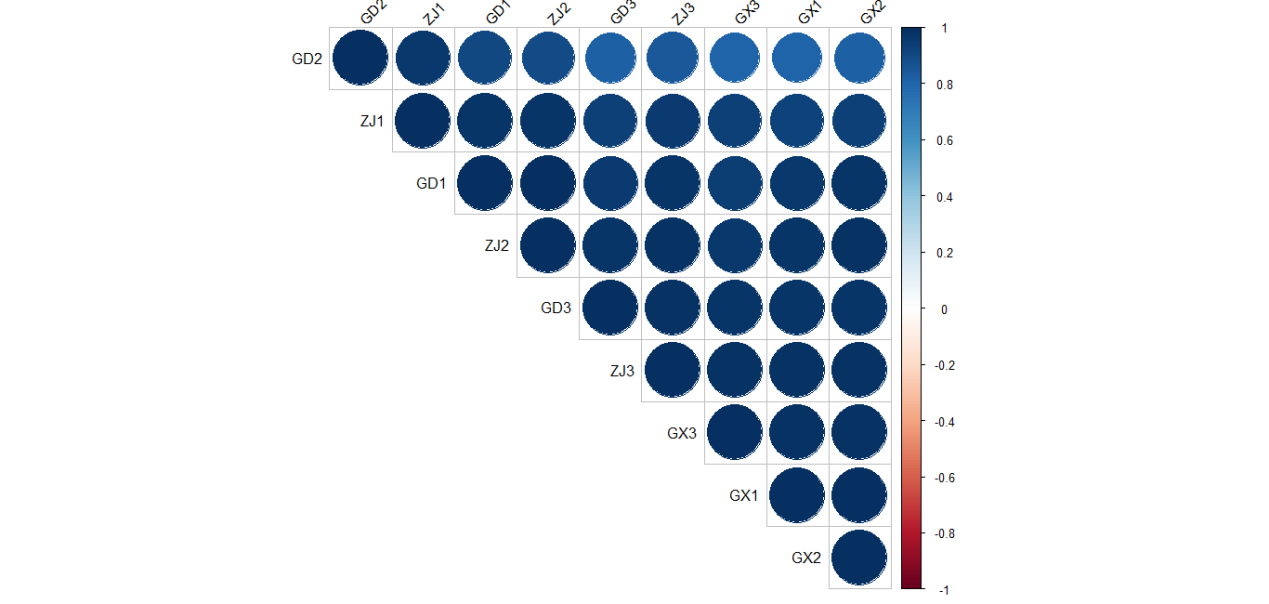


Figure 2S The result of correlation analysis


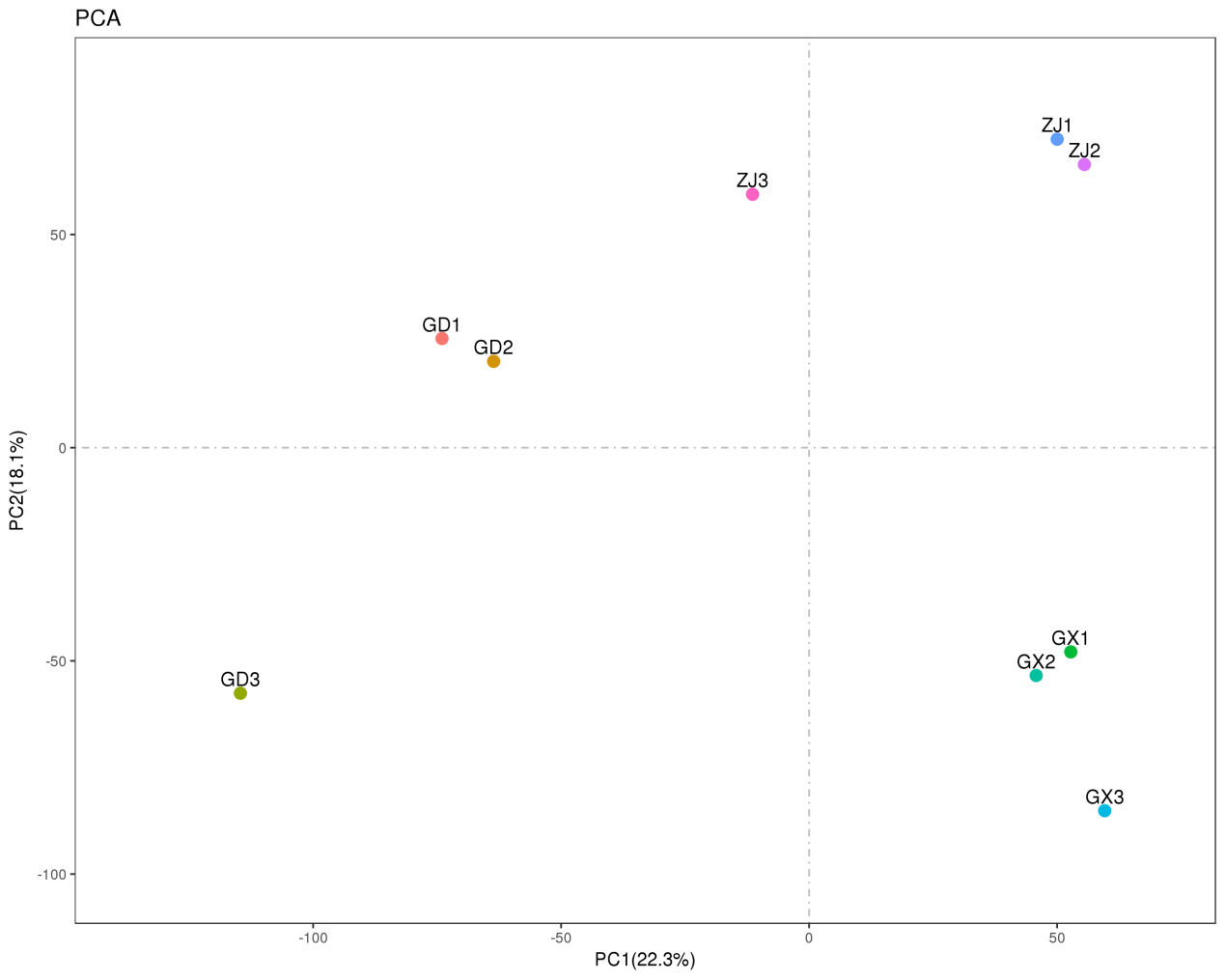


Figure 3S The result of PAC analyses (GD, ZJ and GX were short for Guangdong, Zhejiang and Guangxi provinces)


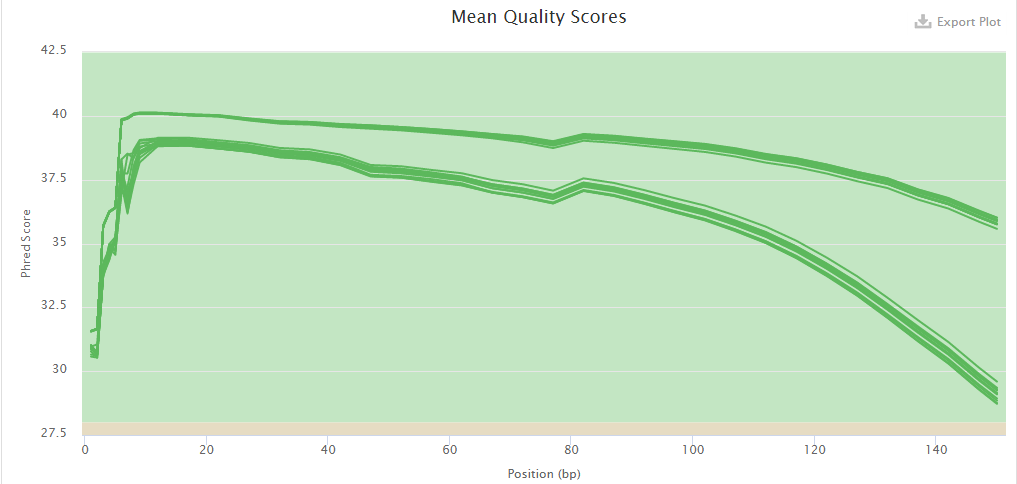


Figure 4S FASTQC graph


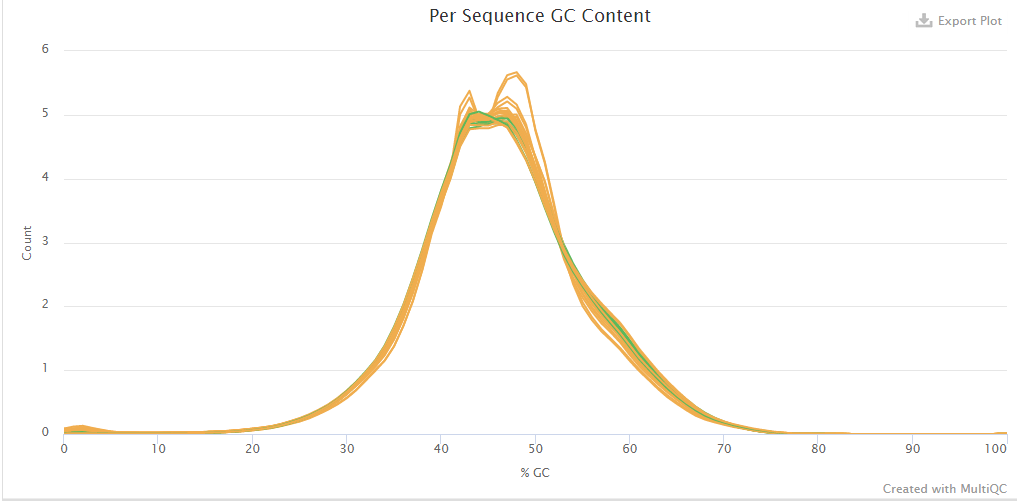


Figure 5S GC content


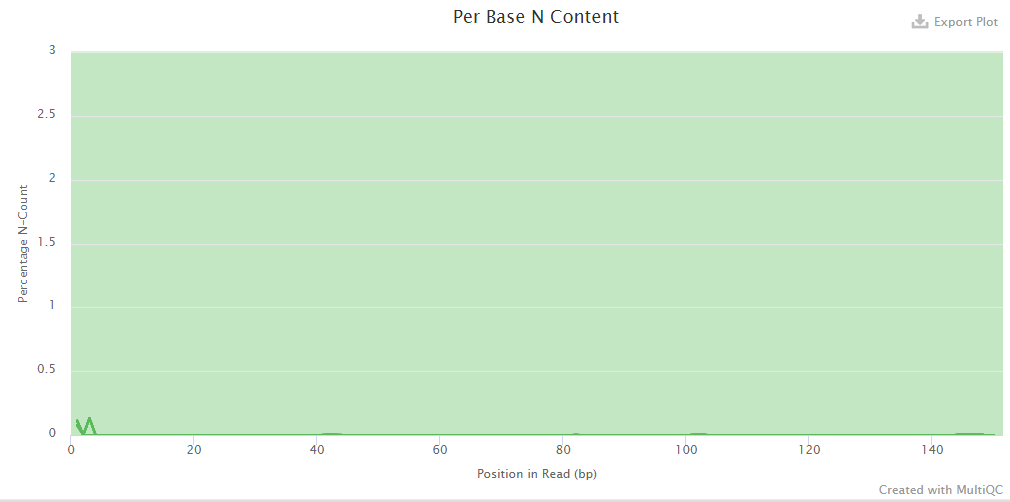


Figure 6S Per base N content


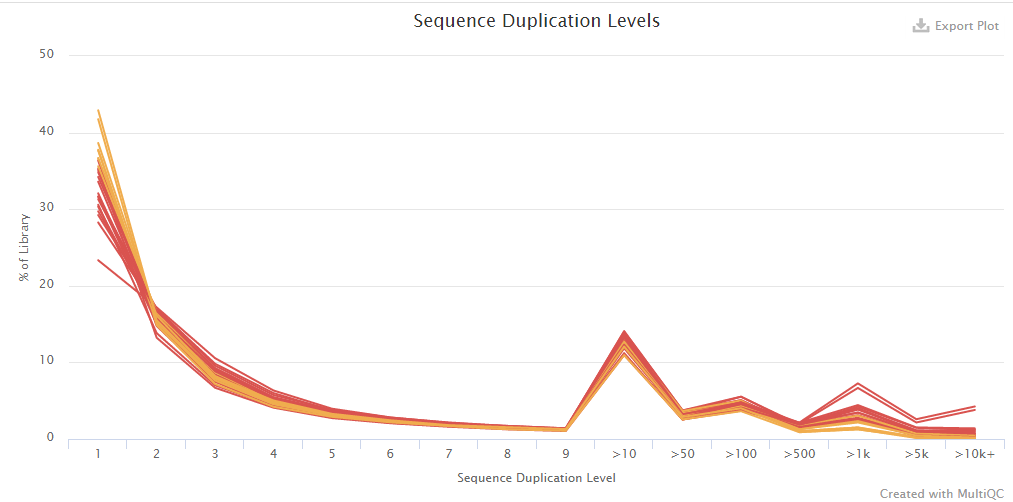


Figure 7S Sequence Duplication Level


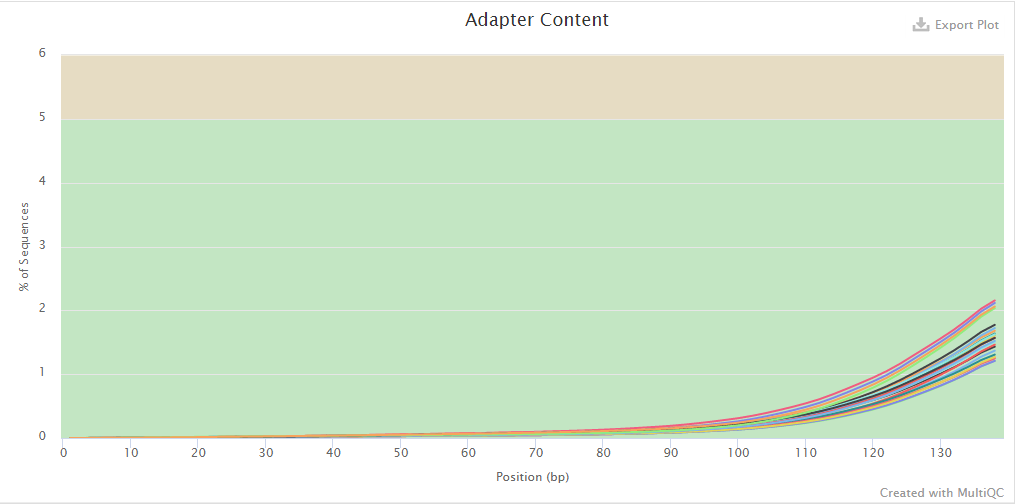


Figure 8S Adapter content
